# Relationship between sleep duration and Framingham cardiovascular risk score and prevalence of cardiovascular disease in Koreans

**DOI:** 10.1097/MD.0000000000007744

**Published:** 2017-09-15

**Authors:** Eui Im, Gwang-Sil Kim

**Affiliations:** a Division of Cardiology, Department of Internal Medicine, Yongin Severance Hospital, Yonsei University College of Medicine, Yongin; b Division of Cardiology, Department of Internal Medicine, Sanggye Paik Hospital Inje University College of Medicine, Seoul, Republic of Korea.

**Keywords:** cardiovascular disease, Framingham cardiovascular risk score, sleep duration

## Abstract

Studies have shown sleep duration to be related to the prevalence of metabolic syndrome and hypertension. However, whether sleep duration is associated with cardiovascular disease (CVD) risk and the prevalence of CVD irrespective of conventional CV-risk factor, such as diabetes mellitus, obesity, and metabolic syndrome, has not been well established for the Korean population.

A total of 23,878 individuals aged 18 years or older from the 2007–2010 Korean National Health and Nutrition Examination Survey were analyzed. We evaluated the relationship between sleep duration and CV-event risk using the Framingham Cardiovascular Risk Score (FRS; ≥10% or ≥20%) and the prevalence of CVD.

After adjusting for traditional risk factors of CVD, a short sleep duration (≤5 hours) yielded odds ratios (OR) of 1.344 (95% confidence interval [CI] 1.200–1.505) for intermediate to high risk and 1.357 (95% CI, 1.140–1.614) for high risk. A long sleep duration (≥9 hours) was also associated with both intermediate to high (OR 1.142, 95% CI 1.011–1.322) and high cardiovascular FRS (OR 1.276, 95% CI 1.118–1.457).

Both short and long sleep durations were related with high CVD risk, irrespective of established CVD risk, and a short sleep duration was associated with a higher prevalence of CVD than an optimal or long sleep duration.

## 1 Introduction

Sleep duration is associated with adverse health outcomes, such as diabetes, impaired glucose tolerance, hypertension, and obesity.^[1–4]^ A previous study in Koreans showed that less than 5 hours of sleep is associated with the prevalence of hypertension.^[4]^ Moreover, the same sleep duration was reported as a risk factor for development of cardiovascular disease (CVD).^[5–7]^ Nevertheless, studies on the relationships between both future CV-event risk and CVD prevalence and sleep duration in Asian general populations are scarce. Additionally, there is also debate as to whether both short and long sleep durations are associated with CVD according to race and age.

Framingham Risk Score (FRS) is the most popular, global risk method for estimating 10-year cardiovascular event risk,^[8]^ and previous studies have shown that it is applicable in Asian population.^[9,10]^ Therefore, in the present study, we aimed to determine whether there is an independent relationship between sleep duration and CV-event risk using FRS and the prevalence of CVD, including stroke and ischemic heart disease, in a representative Korean population using data from the nationwide Korea National Health and Nutrition Examination Survey (KNHANES).

## 2 Materials and methods

### 2.1 Study population

In this study, data collected through the KNHANES from 2007 to 2010 were used. The target population of the KNHANES included all noninstitutionalized Korean civilians of at least 1 year of age. A stratified multistage probability sampling design was used. The participants were selected from sampling units predicated on geographical area, sex, and age, determined using household registries. Each population group was assigned a weighted value based on geographical and demographic characteristics to allow estimates to be calculated for the entirety of the Korean population. Nurses were trained to record anthropometric measurements, collect blood, measure blood pressure, and administer questionnaires. The questionnaires used in this study included questions that addressed the demographic, socioeconomic, dietary, and medical history of each respondent.^[11]^

After excluding 9882 individuals younger than 18 years of age and 1611 without information on sleep duration, 23,878 participants, consisting of 10,209 males (42.8%) and 13,669 females (57.2%), were included in our final analysis. The Institutional Review Board of the Korea Centers for Disease Control and Prevention approved the survey protocol, and all participants provided written informed consent. The study did not require any ethics approval, because the KNHANES data are available for public access.

### 2.2 Sleep duration assessment

Sleep duration was assessed using the following question: “How many hours do you sleep a day?” The duration of sleep was categorized as less than 5 hours, 6–8 hours, or more than 9 hours, in accordance with previous studies.^[12,13]^

### 2.3 Blood pressure and resting heart rate measurements

Blood pressure (BP) was measured with a mercury manometer with the subjects in a sitting position, and the average of 2 blood pressure readings was used for analysis.

The radial pulse was measured for 15 seconds, after resting for 5 minutes in a sitting position, and then multiplied by 4 and used as a resting heart rate (beats/min). In the case that the participant had an irregular pulse, a slow pulse (<15 beats), or a rapid pulse (> 26 beats), the pulse was measured again for a whole minute.

### 2.4 Framingham Risk Score calculation and cardiovascular risk stratification

FRS was calculated based on risk factors, including sex, age, total cholesterol, high density lipoprotein (HDL) cholesterol, systolic blood pressure, treatment for hypertension, smoking, and diabetes status.^[8]^ The Framingham risk groups (taking all risk factors into account) were defined by risk percentages (low < 10%, intermediate 10–20%, high >20%) and obtained only in patients without history of CVD, including ischemic heart disease and stroke.

### 2.5 Definitions of obesity, diabetes, hypertension, and metabolic syndrome

The International Obesity Task Force (IOTF) and World Health Organization (WHO) regional office for the Western Pacific region recommend defining obesity in Asians as a BMI of ≥25 kg/m^2^. Subsequently, the Korean Society for the Study of Obesity (KSSO) adopted this definition.^[14]^ Thus, subjects were classified as obese if their BMIs were ≥ 25 kg/m^2^ according to the standards of the IOTF, WHO, and KSSO.

Diabetes was defined if the patient met one of the following criteria: previous diagnosis of diabetes; current medication of oral hypoglycemic agent or insulin; and glycated hemoglobin (HbA1C≥6.5%)

Participants who had previously been diagnosed with hypertension were taking antihypertensive drugs, or had a systolic blood pressure (SBP) of ≥140 mm Hg or a diastolic blood pressure (DBP) of ≥90 mm Hg were classified as hypertensive.

The modified National Cholesterol Education Program's Adult Treatment Panel III (NCEP ATP III) Asian criteria for metabolic syndrome were used, requiring 3 or more of the following criteria: waist circumference (WC) ≥90 cm for male subjects and ≥85 cm for female subjects ^[15]^; triglyceride level ≥150 mg/dL; HDL-cholesterol level ≤40 mg/dL for male subjects and ≤50 mg/dL for female subjects; blood pressure ≥130/85 mm Hg; and fasting glucose level ≥100 mg/dL.

### 2.6 Statistical Analyses

All data are expressed as numbers and percentages (%), medians (interquartile range), or means ± standard deviation. Continuous variables were compared using an independent *t* test or 1-way analysis of variance (ANOVA). If continuous variables did not show normal distribution, we performed the Kruskal-Wallis test instead of ANOVA. Categorical variables were compared using the chi-square test or Fisher exact test. The odds ratios (ORs) for CVD risk according to sleep duration were calculated using logistic regression. The multivariable-adjusted model was adjusted for known confounding factors, including age, sex, hypertension, diabetes, obesity, and metabolic syndrome. *P*-values < 0.05 were considered statistically significant. The Statistical Package for the Social Sciences (SPSS, version 19; SPSS, Chicago, IL) was used to conduct all analyses.

## 3 Results

Table 1 shows the baseline characteristics of the participants according to their sleep duration. Short (≤5 hours), optimal (6–8 hours), and long (≥9 hours) durations comprised 15.3%, 75.1%, and 9.2% of the study population, respectively. Older and female subjects were more prevalent in the short sleep duration group. BMI and WC were the highest in the short duration group. Both systolic and diastolic blood pressures were the highest in the short duration group; the resting heart rate was highest in the long duration group. Underlying disease, such as DM and dyslipidemia, were also highest in the short duration group. Serum concentrations of HbA1_C_ were highest in the long sleep duration group, and the HOMA IR index was higher in both short and long duration groups than the optimal group.

**Table 1:**
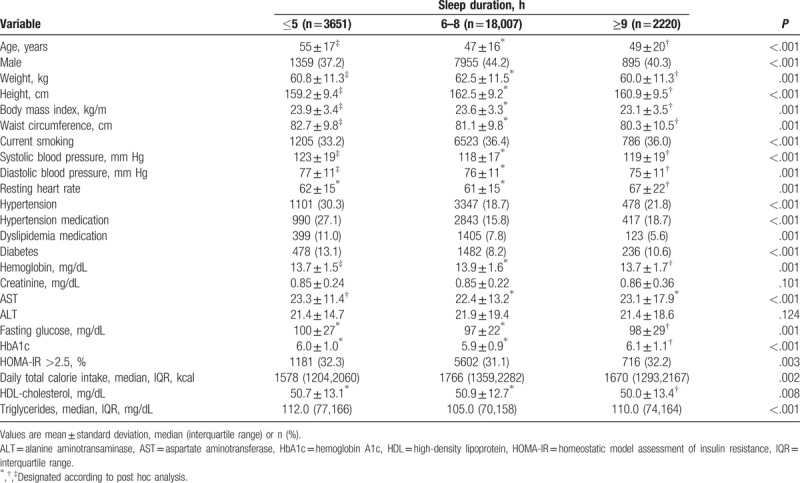
Characteristics of patients according to sleep duration.

The associations between conventional CV-risk factors, prevalence of CVD, and sleep duration are summarized in Table 2. General and abdominal obesity were more prevalent in the short duration group, and the prevalences of stroke and ischemic heart disease were the lowest in the optimal duration group. Both intermediate to high and high CVD risks calculated by the Framingham Cardiovascular Risk Score were highest in the short duration group. The ORs for the 10-year CVD event risk according to sleep duration are shown in Table 3. After adjustment for conventional risk factors for CVD, including age, metabolic syndrome, exercise, smoking, and general obesity, both a short sleep duration (OR, 1.344; 95% confidence interval [CI], 1.200–1.505; *P* < .001) and long duration (OR, 1.142; 95% CI, 1.011–1.322; *P* = .048) remained significant predictors for 10-year intermediate to high CVD event risk, and this trend was equally demonstrated for the prediction of 10-year high CVD event risk.

**Table 2:**
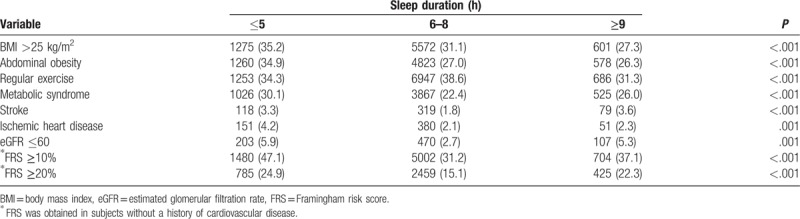
Incidence of metabolic and cardiovascular diseases according to sleep duration.

**Table 3:**
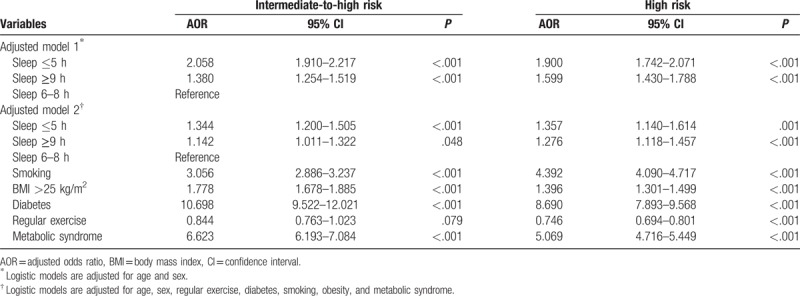
Ten-year cardiovascular event risk according to sleep duration.

**Figure 1:**
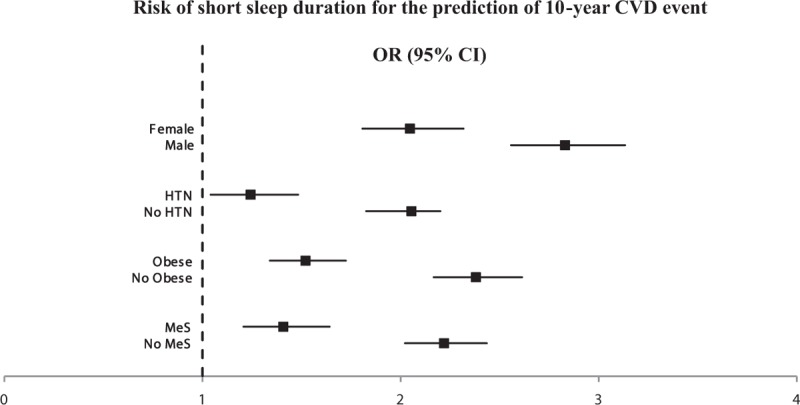
Short sleep duration is a significant predictor of the occurrence of CVD irrespective of sex, hypertension, general obesity, and metabolic syndrome. CVD = cardiovascular disease.

The ORs for the prevalence of CVD were also higher in the short and long sleep duration groups than the optimal duration group. After adjustment for conventional risk factors of CVD, including age, sex, smoking, regular exercise, obesity, and metabolic syndrome, the OR of the short sleep duration group was 1.249 (95% CI, 1.060–1.472; *P* = .001) with statistical significance (Table 4), and this trend was noted irrespective of sex. Figure 1 shows that a short sleep duration was a significant predictor of the occurrence of CVD irrespective of sex, hypertension, general obesity, and metabolic syndrome.

**Table 4:**
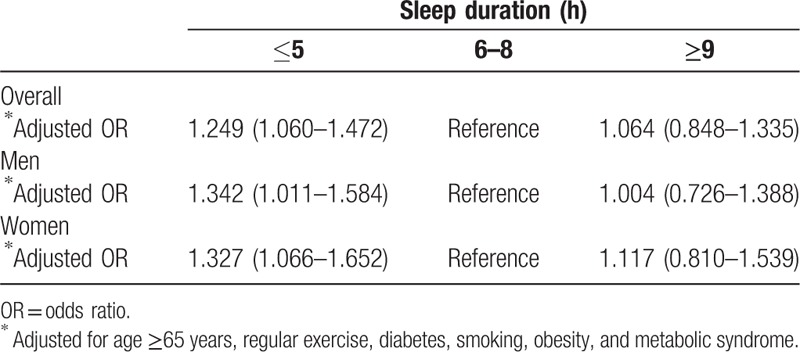
Prevalence of cardiovascular disease (stroke, ischemic heart disease) according to sleep duration.

## 4 Discussion

The main findings of this study were as follows: (1) both a short and long sleep duration were positively associated with FRS-determined 10-year CVD-event risk independent of conventional CVD risk factors, such as age, sex, exercise, metabolic syndrome, smoking, and general obesity; (2) a short sleep duration was associated with the prevalence of CVD without sex difference. Overall, these data suggest that a short sleep duration is associated with a higher prevalence of history of CVD and increases the risk of CV-related events in patients without history of CVD.

CVD is a leading cause of morbidity and mortality worldwide. Modifiable life style factors, such as diet, physical activity, obesity, cigarette smoking, and sleep problems, have been shown to be associated with risk of CVD.^[16]^ Epidemiological studies have reported significant associations between sleep duration and cardio-metabolic risk factors, including hypertension, diabetes, and metabolic syndrome.^[1,2,13,17]^ In recent research, the recommended hypertension treatment rate has increased significantly in Koreans who sleep less than 5 hours. More importantly, the increased risk associated with sleep duration has been found to have translated into the prevalence of overt CVD.^[18,19]^ In Japanese individuals, both sleep durations of 4 hours or less and 10 hours or longer were associated with increases in all causes of mortality in a community-based cohort study, which enrolled about 98,000 persons aged 40 to 79 years.^[20]^ In line with previous studies, our results indicated that a short sleep duration is associated with both general and abdominal obesity, as well as higher prevalences of hypertension, dyslipidemia, diabetes, and metabolic syndrome. Although the mechanisms of these associations are not fully understood, several previous studies have suggested that short sleep durations are related to CVD risk via the following: (a) reduced leptin and elevated ghrelin that could increase appetite,^[21]^ (b) increased cortisol and growth hormone levels that facilitate central and peripheral disturbances and impairment of carbohydrate tolerance,^[22]^ and (c) mediating inflammatory reactions that might be associated with insulin resistance.^[23]^ In our data, central obesity, insulin resistance, and dyslipidemia were highest in the short duration group, although total calorie intake was lowest, and this result might be associated with the hormonal effects mentioned above. Another possible mechanism is poor adrenal signaling or adrenal fatigue due to lack of sleep. Persistent sleep deprivation might cause chronically elevated concentrations of adrenergic hormone and insensitivity of adrenergic receptor. This might be related with CVD risk, since previous studies showed that increased circulating catecholamines and decreased adrenergic receptor responsiveness are associated with abnormal heart function.^[24–26]^

In the present study, the ORs for CV-risk using FRS also increased in the short duration group after adjusting for several factors, such as age, sex, smoking, obesity, diabetes, and metabolic syndrome. Also, in our data, long sleep duration was associated with higher risks of CVD-event than the optimal group, in line with previous studies.^[20,27]^ Abnormal lipid profiles and elevated markers of inflammation have been reported as a possible mechanism in individuals who sleep for a long duration.^[28,29]^ Some previous studies have reported that sleep-related problems are more strongly associated with poor outcomes in women than in men, and these discrepancies were explained to suggest that women tend to have higher rates of insomnia, lower sleep quality, and differences in hormonal action (serotonin and melatonin).^[23,30–32]^ However, we noted no sex difference or difference of sleep duration effect on CV-risk according to the history of hypertension, obesity, and metabolic syndrome in our study.

We also examined whether sleep duration is associated with the prevalence of CVD. In the USA data, long sleep duration was associated with stroke, especially in older people.^[19]^ In a Japanese population, both short and long sleep duration was associated with mortality from CVD and prevalence of CVD ^[20]^; however, other Japanese researchers reported that long sleep duration was associated with higher risk of CVD among older adult patients.^[27]^ In our data, only short duration sleep was associated with the prevalence of CVD in both sexes, and it was consistent after adjusting for age, physical activity, obesity, and metabolic syndrome.

To our knowledge, this is the first study to have examined both prevalence of CVD and future CV-risk without history of CVD in the Korean population. In the present study, we found that both short and long sleep duration is associated with higher risk of 10-year CV-risk and that only short duration is associated with the prevalence of CVD.

Some limitations should be considered in the interpretation of this study. First, it used a cross-sectional design, which limited the ability to detect causal relationships. Therefore, the cause-and-effect relationships of sleep duration with prevalence of CVD or the risk of CV-event cannot be precisely inferred, although this study included a representative sample of the general Korean population and made efforts to adjust for confounding factors using multiple logistic regression analysis. Second, the data were based on a national cross-sectional survey, and some parameters, including blood pressure and heart rate, were measured only once. Thus, such parameters may not reflect the usual condition of the participants fully. There was also limiting data on diseases that could possibly affect the incidence of CVD, such as respiratory disease. Third, we did not assess the quality of sleep, such as snoring, timing of sleep, wakening after sleep onset, all of which may affect our results. Finally, sleep duration was determined by a self-questionnaire, not by a more objective method, such as polysomnography; thus, we could not exclude recall and information bias.

In conclusion, both short (≤5 hours) and long sleep (≥9 hours) durations were found to be related with higher CVD risk irrespective of established CVD risk factors, and the prevalence of CVD was associated with short sleep duration in Koreans.
